# Successful Nonoperative Management of Spontaneous Splenic Hematoma and Hemoperitoneum due to CMV Infection

**DOI:** 10.1155/2012/328474

**Published:** 2012-11-28

**Authors:** Georgios Lianos, Eleftheria Ignatiadou, Christina Bali, Haralampos Harissis, Christos Katsios

**Affiliations:** Department of Surgery, University Hospital of Ioannina, St. Niarchou Avenue, 45110 Ioannina, Greece

## Abstract

*Introduction*. Spontaneous splenic hematoma or splenic rupture due to CMV infection in immunocompetent adults is rare and life-threatening. *Case Report*. Herein we report a rare case of spontaneous splenic hematoma and hemoperitoneum due to CMV infection in a 23-year-old Caucasian male in whom conservative management was successful. *Conclusion*. Spontaneous splenic hematoma and spontaneous splenic rupture are extremely rare conditions during primary CMV infection. Though rare, they must be always considered by the operating surgeon, because any misinterpretation may result in unfavorable outcomes.

## 1. Introduction

 Human cytomegalovirus is a member of the herpes family of viruses and undergoes latency after primary infection [1, 2]. The primary infection is diagnosed by a strongly positive CMV IgM antibody test result or CMV IgG seroconversion. Spontaneous splenic rupture or subcapsular splenic hematoma is really an uncommon condition in primary CMV infection [3]. The management of these complications has been a matter of debate during the last years [4]. Although splenectomy is the appropriate treatment for hemodynamically unstable patients, it seems that nonoperative management in selected patients is nowadays considered the gold standard of care [5, 6].

## 2. Case Report

 A 23-year-old Caucasian male was admitted to the emergency department of our hospital due to severe left upper quadrant abdominal pain. His medical history was free, and no recent trauma was reported. Clinical examination revealed no pyrexia, heart rate at 90 per minute, and normal blood pressure. Upon physical examination, upper abdominal tenderness was revealed. On auscultation, abdominal sounds were present. Rectal examination showed an empty rectum.

**Figure 1:**
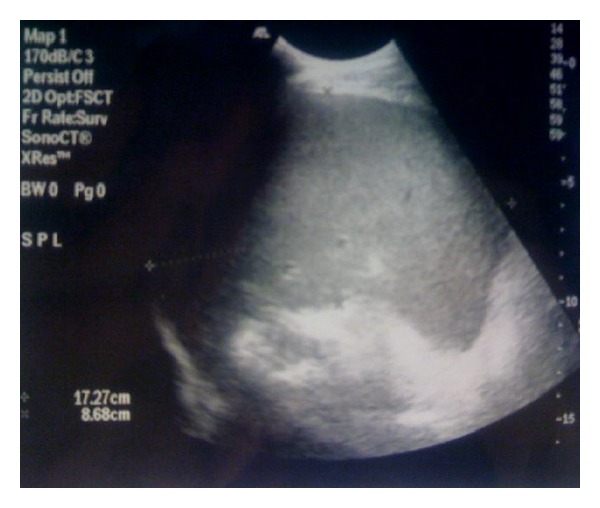
Abdominal ultrasound showed splenomegaly (17,27 × 8,7 cm) and free fluid in the left iliac fossa.

**Figure 2:**
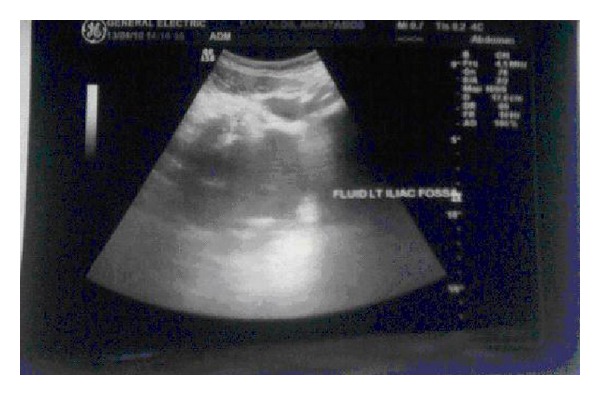
Abdominal ultrasound showed splenomegaly (17,27 × 8,7 cm) and free fluid in the left iliac fossa.

**Figure 3:**
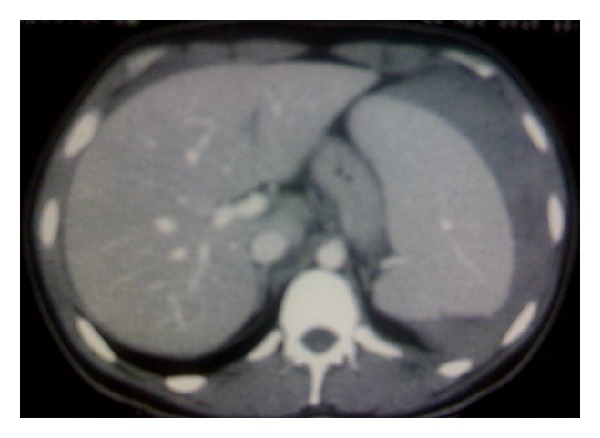
Urgent abdominal computerized tomography (CT) showed an enlarged spleen and splenic hematoma with the presence of free intraperitoneal fluid.

The emergent laboratory tests revealed the following: WBC 24350/mm^3^, hemoglobin 14,2 g/dl, platelets 278000/mm^3^, c-reactive protein 17 mg/dl, Tbil 1,9 mg/dl, Dbil 0,28, ast 27 IU/L, alt 42 IU/L, and creatinine and electrolytes were normal. An abdominal ultrasound showed splenomegaly (17,27 × 8,7 cm) and free fluid in the left iliac fossa and in pelvis (Figures 1 and 2). The urgent abdominal computerized tomography (CT) confirmed an enlarged spleen and showed a splenic hematoma with the presence of free fluid in the paracolic gutters and pelvis. There was a hyperdense component within the free fluid, indicating hemoperitoneum (Figure 3).

**Figure 4:**
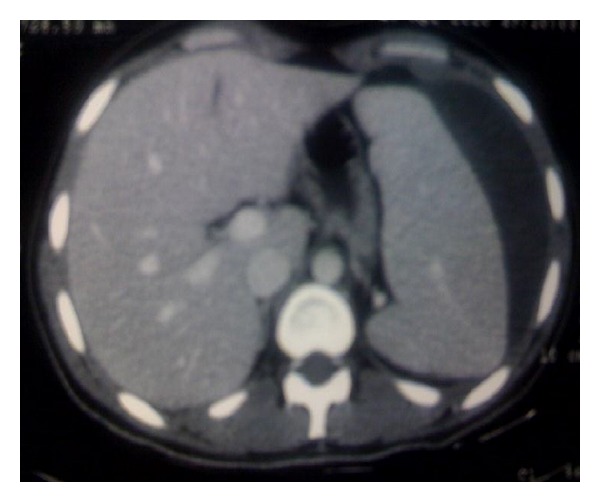
CT, two weeks after patient's admission, showed the same splenic hematoma.

The patient was managed conservatively because of his hemodynamic stability. The diagnosis of primary CMV infection was made with positive IgM anti-CMV antibodies. He was under close monitoring and surgical supervision. He was hemodynamically stable all the time, and two weeks later a new CT of the abdomen was arranged. CT demonstrated the same splenic hematoma and a little resolution of the peritoneal effusion (Figure 4). After a period of prolonged bed rest (twenty days), the patient was discharged from the hospital with the advice to avoid extreme sports and activities. Five months later, followup with ultrasound examinations showed progressive resolution of the splenic hematoma and complete resolution of the peritoneal effusion.

## 3. Discussion

It is reported that spontaneous splenic rupture is extremely rare (0,1%–0,5%) [7, 8]. Clinical presentation includes severe upper abdominal pain with guarding and tenderness. Crucial for prompt surgical intervention is the early diagnosis of splenic rupture. The pathologies which lead to spontaneous splenic hematoma and splenic rupture include hematological malignancies (acute leukemia, chronic leukemia), splenic tumors and metastases, infections (Epstein-Barr virus, malaria, and CMV), amyloidosis, drug-induced disorders, and disorders in pregnancy [9].

The mechanism for the spontaneous hematoma and the splenic rupture is not yet fully clear. However, it seems that it consists in increased intrasplenic tension caused by cellular hyperplasia and vascular occlusion caused by reticular endothelial hyperplasia. This leads to subcapsular hemorrhage, formation of subcapsular hematoma, and rupture of the splenic capsule [10].

Spontaneous subcapsular splenic hematoma with hemoperitoneum, splenic contusions-lacerations, and complete spontaneous splenic rupture are rare and life-threatening complications of primary CMV infection in young immunocompetent adults. To our knowledge, only few reports on splenic “surgical” involvement during primary CMV infection treated conservatively have been published to date.

In order to explore this, we conducted a PubMed search using the key words “splenic hematoma,” “CMV infection,” “conservative management,” and “splenic rupture.” We found only five cases, including our case, of “surgical” splenic involvement during primary CMV infection in immunocompetent adults. These patients were treated successfully with nonoperative management [11, 12] (Table 1).

In case of hemodynamic instability, it is obvious that splenectomy is the treatment of choice. When, however, the patient is hemodynamically stable, as in our case, the non-operative management is a safe option with the patient under close monitoring and surgical supervision [13–15]. Recent studies showed that splenectomy dogma tends to be supplanted by conservative management. Conservative treatment can be successful if appropriate criteria and a close monitoring are applied in selected hemodynamically stable patients. Splenectomy should be reserved only for patients with uncontrollable rupture and hemodynamic instability [16, 17].

## 4. Conclusion

In conclusion, the diagnosis of splenic hematoma or splenic rupture due to CMV infection in young immunocompetent individuals can be very difficult and requires a high suspicion and a lot of awareness on the part of the surgeon. These conditions must be always considered when dealing with left upper abdominal pain in young immunocompetent adults in order to prevent the dramatic consequences of a missed diagnosis.

## Figures and Tables

**Table 1:** Successful conservative management in case of splenic rupture or splenic hematoma due to CMV infection in young immunocompetent individuals.

Reference	Sex	Age	Comorbidities	CMV diagnosis	Spleen lesions
(1) Losada et al., 1997 [11]	Female	30	—	(i) Viruria (ii) IgM	Subcapsular Hematoma

(2) Bellaiche et al., 1998 [12]	Male	22	—	(i) Seroconversion (ii) IgM	Splenomegaly Hemoperitoneum

(3) Maillard et al., 2007 [4]	Male	29	Pyruvate Kinase deficiency	(i) IgM (ii) Positive blood PCR (iii) pp65 antigenemia	Partial splenic rupture

(4) Maillard et al., 2007 [4]	Female	22	Iron deficiency anemia	(i) IgM, seroconversion (ii) Positive blood PCR (iii) pp65 antigenemia	Hemoperitoneum Splenic contusions

(5) Our case (Lianos et al.)	Male	23	—	(i) IgM	Hemoperitoneum Splenic hematoma
